# Time-Homogeneous Markov Modeling of HIV Progression in Patients Receiving Antiretroviral Therapy Treatment in the Ashanti Region, Ghana

**DOI:** 10.1155/cjid/5549653

**Published:** 2025-04-22

**Authors:** Michael Fosu Ofori, Gerald Ohene Agyekum, Michael Arthur Ofori, Samuel Akwasi Adarkwa

**Affiliations:** ^1^Department of Statistical Sciences, Kumasi Technical University, Kumasi, Ghana; ^2^Department of Mathematics, Tarleton State University, Stephenville, Texas, USA; ^3^School of Public Health, University of Memphis, Memphis, Tennessee, USA; ^4^Department of Mathematics, Pan African University Institute for Basic Sciences Technology and Innovation, Nairobi, Kenya

**Keywords:** antiretroviral therapy (ART), disease progression, HIV/AIDS, Markov model, multistate model

## Abstract

The global HIV/AIDS pandemic remains a profound public health challenge, with substantial impacts on mortality and morbidity worldwide. In Ghana, where HIV prevalence persists, understanding disease progression among patients receiving antiretroviral therapy (ART) is crucial. This study, conducted in the Ashanti Region, employs a 5-state continuous-time Markov multistate model to analyze HIV progression based on CD4 cell counts, employing tuberculosis (TB) coinfection as a covariate. A retrospective cohort of 416 patients from St. Martins Catholic Hospital between 2000 and 2019 was studied. Transition intensities, sojourn time and probabilities between CD4 states, and the impact of TB coinfection were evaluated. The results showed that patients with CD4 counts ≥ 500 cells/mm^3^ spent more time before transitioning to lower CD4 levels, indicating the effectiveness of ART in controlling the disease at this level. However, the transition from 200–350 cells/mm^3^ to death was more likely than recovery to CD4 counts ≥ 500 cells/mm^3^, indicating the increased risk of mortality once CD4 counts drop significantly. TB coinfection did not significantly alter these transition probabilities, which may be due to the effective management of both HIV and TB in this cohort, emphasizing the need for integrated care strategies. This study emphasizes the importance of tailored interventions to manage HIV/AIDS effectively, particularly in regions with high disease burden. It is recommended that initiating treatment quickly can help maintain higher CD4 counts and improve survival.

## 1. Introduction

Since the identification of human immunodeficiency virus (HIV)/acquired immunodeficiency syndrome (AIDS), clinical researchers and policymakers have been encountering challenges in addressing the global HIV pandemic and the resulting development of AIDS. Having claimed about 40.4 million lives worldwide, 39.0 million people continue to live with HIV. The African Region bears a significant burden, with 25.6 million cases reported [1]. In the year 2022 alone, there were reports of 630,000 deaths attributed to HIV and its related complications, while 1.3 million individuals contracted HIV worldwide [1].

Ghana has witnessed a steady increase in HIV/AIDS cases since the first reported case in 1986. In 2022, about 17,000 cases were reported, resulting in a national prevalence rate of 1.7% among adults aged 15 to 49, with an estimated 350,000 people living with HIV (PLWHIV) in the country. Tragically, 9400 individuals lost their lives due to HIV/AIDS in the same year [2]. In 2019, regional HIV/AIDS prevalence varied across Ghana, ranging from 2.47% in the Greater Accra Region (the highest) to 0.24% in the North East region. The Ashanti Region recorded a prevalence of 1.94% [3].

Tuberculosis (TB) is a bacterial infection that can spread from person to person, mainly affecting the lungs but also potentially impacting other areas of the body. It is caused by the *Mycobacterium tuberculosis* bacterium, which transmits through the air when someone with the infection coughs, sneezes, or spits. In 2019, there were approximately 10 million reported cases of TB globally, resulting in about 208,000 deaths among individuals coinfected with HIV. Out of these cases, 7.1 million (71%) were documented in national TB programs, leaving approximately 2.9 million (29%) of individuals with undetermined disease trajectories [4].

The synergistic relationship between TB and HIV poses a significant threat to public health and economic development, especially in sub-Saharan Africa [5]. HIV infection is marked by a gradual depletion of CD4+ T-cells, which play a crucial role in the immune system. This decline increases the risk of reactivating latent TB by 20-fold [6], and studies have shown a significant rise in the likelihood of developing active TB soon after the initial HIV infection [7]. Among the estimated 400,000 global annual deaths from TB in individuals coinfected with HIV, approximately 51% occur in sub-Saharan Africa [8].

Ghana is categorized as a high-risk country for TB-HIV coinfection by the World Health Organization (WHO) due to its significant prevalence of both diseases. In 2015, there were about 9900 new TB cases identified among individuals living with HIV in Ghana, translating to a rate of 36 cases per 100,000 people [9]. Research in Ghana reveals that the coinfection of TB and HIV is becoming an increasingly significant challenge, with over 14% of TB cases now associated with HIV [9, 10]. Ghana continues to face significant challenges in managing TB and HIV coinfections, particularly among patients with multidrug-resistant TB, where treatment outcomes and antiretroviral uptake remain critical.

Despite advancements in combating HIV/AIDS through interventions like antiretroviral therapy (ART), challenges persist, including the emergence of drug resistance [11]. ART has been essential in significantly reducing HIV/AIDS-related mortality and morbidity rates. The primary goal of ART for PLWHIV is to suppress HIV/AIDS replication and restore immune function [12]. Particularly in sub-Saharan Africa, where the burden of the disease tends to be most acute, ART serves as the primary means to manage and alleviate the health crises caused by HIV/AIDS infection [13]. These drugs function by inhibiting the vital enzymes necessary for HIV replication [14]. CD4 is used as a prognostic marker of disease progression and for categorization of HIV infection into different states (states 1, 2, 3, and 4) [14]. The CD4 cell count serves as a crucial indicator for assessing PLWHIV and is utilized as a prognostic marker to evaluate disease progression. Besides its role as a primary marker of disease advancement, CD4 is used to categorize diseases into stages.

Previous studies [11, 15–19] have modeled disease progression in patients receiving ART using their CD4 cell counts using different Markov models. Despite advancements in HIV/AIDS research, there remains an unexplored area such as the evaluation of how HIV patients respond to ART. In this paper, we investigate the intensities of HIV patients on ART treatment as they transit from one state to another using Markov models based on CD4 cell counts by including TB coinfection as a covariate.

## 2. Materials and Methods

### 2.1. Study Design

A retrospective cohort of 416 patients identified with HIV and initiated on ART between 2000 and 2019 at St. Martins Catholic Hospital in the Amansie South District of the Ashanti Region was studied. The sample size of 416 patients was determined through power analysis. Using a moderate effect size of 0.25, alpha level of 0.05, and power of 0.80, a minimum sample of 400 participants was required to detect statistically significant associations between the study variables. The final sample size of 416 provided a small buffer to ensure adequate statistical power while remaining feasible within resource constraints. The sampling frame consisted of all 1842 HIV-positive patients who initiated ART at St. Martins Catholic Hospital between January 1, 2000, and December 31, 2019, as documented in the hospital's comprehensive HIV/AIDS registry. The period from 2000 to 2019 was strategically selected for several reasons. The year 2000 marked the beginning of systematic ART provision at St. Martins Catholic Hospital following Ghana's national HIV treatment guideline implementation. This 19-year timeframe encompassed several important transitions in HIV care, including the introduction of first-line regimen changes in 2006 and 2013, the implementation of “Test and Treat” protocols in 2016, and the adoption of dolutegravir-based regimens in 2018. The patients were selected by employing simple random sampling, ensuring that everyone in the population had an equal chance of being included. This method minimized bias and aimed to create a representative subset reflective of the broader group. Throughout the study period, all 416 patients were monitored continuously until either recovery or death, with no losses to follow-up.

### 2.2. Overview of Multistate Models

According to Reddy [20], in a Markov model, the transition to a future state depends solely on the current state of the process, with no consideration of the history preceding that state. That is, past events are irrelevant for predicting future transitions.

A multistate model is a model for continuous-time stochastic process say {*Z*(*t*) : *t* ∈ *T*} which tends to occupy one of a few possible states [21]. The evaluation of the survival outlook is a common focus in medical research studies when patients have a condition that spreads from one state to another. Only two states are directly modeled by standard survival models: living and dead. Multistate models allow for the direct modeling of disease development when patients are observed to be in different states of health or disease at random intervals, but the times of entering or leaving states are unknown.

Based on the recognized ranges of CD4 count, the five states of HIV disease progression of patients on ART are described as follows in this study. State 1 (CD4 ≥ 500 cells/mm^3^) reflects optimal immune function. Patients in this state are generally healthy and at low risk for opportunistic infections. State 2 (351 cells/mm^3^ > CD4 > 500 cells/mm^3^) signifies moderate severity. State 3 (200 cells/mm^3^ > CD4 > 350 cells/mm^3^) indicates a severe compromise of immune function. Patients become increasingly vulnerable to opportunistic infections. State 4 (CD4 < 200 cells/mm^3^) represents critical severity, with patients experiencing severe immune dysfunction and heightened risk of life-threatening infections. State 5 signifies death, marking the end stage of HIV disease progression.

**Figure 1:**
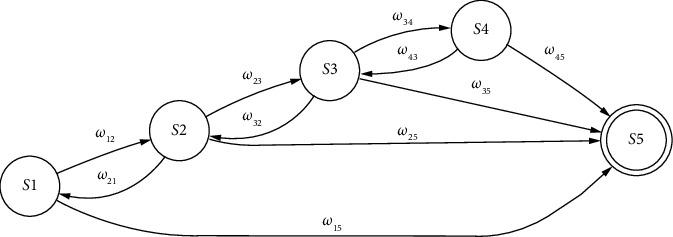
Transition diagram for HIV patients undergoing antiretroviral therapy treatment.

Using these five states, the progression of HIV positive individuals on treatment is defined by the state diagram shown in Figure 1. The arrows in the diagram show possible transitions between the five states defined above. Also, state 5 is an absorbing state, meaning there are no transitions out of this state. As HIV advances within a person's body, there is a chance that the individual may remain in a consistent state during consecutive visits.

### 2.3. Transition Intensities

Suppose there are p HIV patients on ART being studied; a patient may move in a 5-state Markov model that has a discrete state space, *S* = {1,2,3,4,5}. States 1, 2, 3, and 4 represent transient states, while state 5 serves as the absorbing state.

If *Z*(*t*) = *u* is the state of a patient at any time *t*, then the intensity with which the patient moves to state *v* during the interval (*t*, *t* + *δt*) is defined as

(1) $$\begin{array}{c} \omega_{uv}\left(t\right)=\underset{\delta t⟶0}{\lim}\frac{p\left(Z\left(t+\delta t\right)=v\left|Z\right.\left(t\right)=u\right)}{\delta t},\text{ for }u,v=1,2,3,4,5. \end{array}$$

The transition intensity matrix is given by

(2) $$\begin{array}{c} A\left(t\right)=\left[\begin{array}{cccccc} \omega_{11} & \omega_{12} & \omega_{13} & \omega_{14} & \ldots & \omega_{1p}\\ \omega_{21} & \omega_{22} & \omega_{23} & \omega_{24} & \ldots & \omega_{2p}\\ \omega_{31} & \omega_{32} & \omega_{33} & \omega_{34} & \ldots & \omega_{3p}\\ \omega_{41} & \omega_{42} & \omega_{43} & \omega_{44} & \ldots & \omega_{4p}\\ \vdots & \vdots & \vdots & \vdots & \vdots & \vdots \\ \omega_{p1} & \omega_{p2} & \omega_{p3} & \omega_{p4} & \ldots & \omega_{pp} \end{array}\right], \end{array}$$

where *A*(*t*) is the *p* × *p* matrix which represents the transition intensity matrix for a process involving a maximum of p states. This matrix provides the instantaneous rate at which transitions occur from one state to another.

The entry (*u*, *v*) is 0 when there is no possibility of transitioning from state *u* to *v*.

The transition intensity matrix, defined as *P* = (*ω*_*uv*_)_5×5_, has the following properties:

a. ∑_*v*∈*S*_*ω*_*uv*_ = 0 ∀ *u*,
b. *ω*_*u*_ = ∑_*u*≠*v*_*ω*_*uv*_,
c. *ω*_*uu*_ = −∑_*u*≠*v*_*ω*_*uv*_ ∀ *u*,
  where *ω*′*s* are the transition intensities.

Based on (b) and (c) above, we can now modify the *A* matrix defined previously as

(3) $$\begin{array}{c} A=\left[\begin{array}{cc} {-\omega }_{11} & \begin{array}{ccc} \omega_{12} & \omega_{13} & \begin{array}{cc} \omega_{14} & \omega_{15} \end{array} \end{array}\\ \begin{array}{c} \omega_{21}\\ \omega_{31}\\ \begin{array}{c} \omega_{41}\\ \omega_{51} \end{array} \end{array} & \begin{array}{c} \begin{array}{ccc} {-\omega }_{22} & \omega_{23} & \begin{array}{cc} \omega_{24} & \omega_{25} \end{array} \end{array}\\ \begin{array}{ccc} \omega_{32} & {-\omega }_{33} & \begin{array}{cc} \omega_{34} & \omega_{35} \end{array} \end{array}\\ \begin{array}{c} \begin{array}{ccc} \omega_{42} & \omega_{43} & \begin{array}{cc} {-\omega }_{44} & \omega_{45} \end{array} \end{array}\\ \begin{array}{ccc} \omega_{52} & \omega_{53} & \begin{array}{cc} \omega_{54} & {-\omega }_{55} \end{array} \end{array} \end{array} \end{array} \end{array}\right]. \end{array}$$

The transition intensities estimates are used to complete the transition probability matrix [*P*_*uv*_(*t*)]_5×5_, where *P*_*uv*_(*t*) is the probability of HIV/AIDS patients on ART being in state *v* at time *t*, given that the patient was in state *u* at time *s*. The transition probabilities are given by

(4) $$\begin{array}{c} P_{uv}\left(t\right)=P\left(Z\left(t\right)=v\left|Z\right.\left(s\right)=u\right),\;\forall \;u,v\in S,\\ t\geq s,\text{ and}. \end{array}$$

The equation obeys the Chapman–Kolmogorov equation given as

(5) $$\begin{array}{c} P_{uv}\left(t+s\right)=\underset{w\in X}{\sum }P_{uw}\left(s\right)P_{vw}\left(t\right),\;\forall s\;t>0. \end{array}$$

The transition probability matrix is given as

(6) $$\begin{array}{c} P=\left[\begin{array}{cc} p_{11} & \begin{array}{ccc} p_{12} & p_{13} & \begin{array}{cc} p_{14} & p_{15} \end{array} \end{array}\\ \begin{array}{c} p_{21}\\ p_{31}\\ \begin{array}{c} p_{41}\\ p_{51} \end{array} \end{array} & \begin{array}{c} \begin{array}{ccc} p_{22} & p_{23} & \begin{array}{cc} p_{24} & p_{25} \end{array} \end{array}\\ \begin{array}{ccc} p_{32} & p_{33} & \begin{array}{cc} p_{34} & p_{35} \end{array} \end{array}\\ \begin{array}{c} \begin{array}{ccc} p_{42} & p_{43} & \begin{array}{cc} p_{44} & p_{45} \end{array} \end{array}\\ \begin{array}{ccc} p_{52} & p_{53} & \begin{array}{cc} p_{54} & p_{55} \end{array} \end{array} \end{array} \end{array} \end{array}\right]. \end{array}$$

Upon identifying the transition intensities, the transition probabilities can be derived by solving a set of differential equations referred to as Kolmogorov's forward equations, subject to initial conditions. The Kolmogorov's forward equation is expressed as follows:

(7) $$\begin{array}{c} \frac{dP_{uv}\left(t\right)}{dt}=\underset{\forall w}{\sum }P_{uw}\left(t\right)\omega_{wv},\;\forall u,v. \end{array}$$

### 2.4. Model Formulation

The model assumes that within a small-time interval *δt* ∈(*t,t* + *δt*), transitions can occur from any state u (where *u* = 1, 2, 3, or 4) to any state *v* (where *v* = 1, 2, 3, 4, or 5), according to the following rules:

1. The CD4 cell count of a patient is expected to elevate due to the effectiveness of the treatment at a rate of *ω*_*uv*_, where *v* = *u* − 1.
2. A number of patients do not adhere to their treatment therapy, which can lead to a decrease in their CD4 cell count at a rate of *ω*_*uv*_, where *v* = *u* + 1.
3. Across all states *u* = 1,2,3,4, patients may experience mortality at a specified rate of *ω*_*i*5_.
4. A patient may stay in the same state at a rate of *ω*_*uu*_ = −*λ*_*u*_ = −(*ω*_*u*,*u*−1_ + *ω*_*u*,*u*+1_ + *ω*_*u*5_). This is due to the condition that the sum of transition rates originating from any state equals zero.

The above assumptions are represented by the following transition rate matrix *A*(*t*):

(8) $$\begin{array}{c} A\left(t\right)=\left[\begin{array}{ccccc} -\left(\omega_{12}+\omega_{15}\right) & \omega_{12} & 0 & 0 & \omega_{15}\\ \omega_{21} & -\left(\omega_{21}+\omega_{23}+\omega_{25}\right) & \omega_{23} & 0 & \omega_{25}\\ 0 & \omega_{32} & -\left(\omega_{32}+\omega_{34}+\omega_{35}\right) & \omega_{34} & \omega_{35}\\ 0 & 0 & \omega_{43} & -\left(\omega_{43}+\omega_{45}\right) & \omega_{45}\\ 0 & 0 & 0 & 0 & 0 \end{array}\right]. \end{array}$$

After deriving the transition rate matrix, the transition matrix can be determined utilizing Kolmogorov's forward differential equations, as defined in Equation (7). This results in the subsequent differential equations for the Markov jump processes.

For state 1,
  (9) $$\begin{array}{c} \frac{{dP}_{1}\left(t\right)}{dt}=-\left(\omega_{12}+\omega_{15}\right)P_{1}\left(t\right)+\omega_{21}P_{2}\left(t\right). \end{array}$$
For state 2,
  (10) $$\begin{array}{c} \frac{{dP}_{2}\left(t\right)}{dt}=\omega_{12}P_{1}\left(t\right)-\left(\omega_{21}+\omega_{23}+\omega_{25}\right)P_{2}\left(t\right)+\omega_{32}P_{3}\left(t\right). \end{array}$$
For state 3,
  (11) $$\begin{array}{c} \frac{{dP}_{3}\left(t\right)}{dt}=\omega_{23}P_{2}\left(t\right)-\left(\omega_{32}+\omega_{34}+\omega_{35}\right)P_{3}\left(t\right)+\omega_{43}P_{4}\left(t\right). \end{array}$$
For state 4,
  (12) $$\begin{array}{c} \frac{{dP}_{4}\left(t\right)}{dt}=\omega_{34}P_{3}\left(t\right)-\left(\omega_{43}+\omega_{45}\right)P_{4}\left(t\right)+\omega_{43}P_{4}\left(t\right),\\ \frac{{dP}_{5}\left(t\right)}{dt}=\omega_{15}P_{1}\left(t\right)+\omega_{25}P_{2}\left(t\right)+\omega_{35}P_{3}\left(t\right)+\omega_{45}P_{4}\left(t\right). \end{array}$$

There is no transition from state five because it is the absorbing state.

### 2.5. Sojourn Time and Total Length of Stay

In a multistate model, we estimate the average duration that a patient remains in a transient state during a single stay before transitioning to other states. This duration is known as the mean sojourn time. The calculation for the expected sojourn time is as follows:

(13) $$\begin{array}{c} \frac{-1}{\omega_{vv}}, \end{array}$$

where *ω*_*vv*_ is the *v*^th^ diagonal entry of *A*(*t*).

For instance, the mean sojourn time in state 1 is (−1/−0.3996) = 2.5024 as shown in Table 1.

Additionally, there is a focus on estimating the total duration of stay in each transient state, which refers to the expected time a patient spends in each state over the study period [17]. It is important to predict the total time patients undergoing treatment will spend in both favorable and unfavorable states before death. The forecasted durations of time spent in each state *v* between two future time points *t*_1_ and *t*_2_ are calculated using the formula:

(14) $$\begin{array}{c} l_{v}=\int_{t_{1}}^{t_{2}}P_{uv}\left(t\right)dt, \end{array}$$

where *u* represents the initial state of the process, typically set to 1 [16].

## 3. Results

Table 2 reveals that a higher proportion of patients on treatment are males (64.18%) compared to females (35.82%). Additionally, a large majority (83.17%) of the patients are over the age of 45, while only 22.36% are 45 years old or younger. Among the patients, only 3.61% (15 patients) are coinfected with TB, while the majority (96.39%, 401 patients) are not. Furthermore, most patients are single (74.52%), with married (7.21%), divorced (10.58%), and widowed (7.69%) being much less common.

In Table 3, it can be observed that the mortality rate was higher for females (47.6%) compared to males (25.9%), suggesting a gender-based disparity in survival outcomes during ART. The *P* value of 0.001 indicates that the difference in survival rates between males and females is statistically significant, meaning that the difference in survival outcomes is not due to random chance.

In Table 4, it can be observed that all 62 patients who developed TB had CD4 counts below 200. No patients with CD4 counts above 200 developed TB.

Table 1 presents the summary of transitions between states for the whole study period. The decrease in CD4 cell counts is clear from the table as observed in the cases of forward transitions. A total of 389 (121, 21, 148, 43, 56) transitions were observed from lower to higher states. However, a total of 2 backward transitions were seen from high to low states (3 ⟶ 1 and 3 ⟶ 2).

From the estimated intensities in Table 5, it can be observed that patients in state 2 (351–500 CD4 cell count) are about 3 times (0.4873/0.1462) more likely to transition to state 3 (200–350 CD4 cell count) than to transition state 4 (< 200 CD4 cell count). Also, patients in state 3 are about 77 times more likely to die than to transition to state 1 (> 500 CD4 cell count), indicating that death is more probable than recovery.

Results in Table 6 present estimates of the sojourn time, along with their standard errors and lower and upper bounds, for each patient in state *u*. It can be observed that patients in state 1 (CD4 ≥ 500 cells/mm^3^) spend more time in that state before transitioning to other states. This might be attributed to the fact that state 1 is where ART treatment is most effective in controlling the disease, resulting in a longer duration spent in this state. State 1 represents the best state, reflecting a well-functioning immune system with a low risk of opportunistic infections and complications. In contrast, state 5 (death) represents the worst health outcome, signifying the end stage of HIV disease progression.

In Table 7, it can be observed that each patient is predicted to spend 2.5273 years in state 1, 1.6331 years in state 2, 1.009 years in state 3, and 0.2986 years in state 4. For example, results indicate that patients undergoing ART treatment, upon entering a CD4 cell count greater than 500, can anticipate spending approximately 2.5273 years at that level prior to moving to either an increased or decreased count. These findings show that patients under treatment are likely to spend much time in favorable states compared to unfavorable states.

In Table 8, it can be observed that patients in state 1, the highest CD4 count range, will always move to state 2 with 100% probability. This deterministic transition suggests that patients, even those starting with robust immune function, will experience a decrease in their CD4 count over time but remain within a relatively healthy range. Patients in state 2 have a high probability (76.92%) of transitioning to state 3 and a 23.08% probability of transitioning to state 4. This indicates a significant probability of further decline in their immune function, which necessitates close monitoring and potentially intensified treatment to prevent further deterioration. Also, patients in state 3 mostly transition to state 5 (death) with a probability of 95.66%. Small probabilities exist for transitioning to state 1 (1.25%) and state 2 (3.09%), indicating that while there is a slight chance of improvement, the enormous likelihood is a severe outcome. Further, patients in state 4, with the lowest CD4 counts, will always transition to state 5. This indicates a dire prognosis for patients with CD4 < 200 cells/mm^3^, emphasizing urgent need for intensive care and potential adjustments in their treatment regimen.

**Figure 2:**
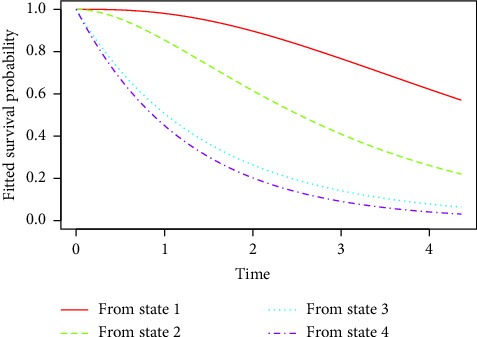
Survival probability by HIV disease stage and treatment duration.

From Figure 2, it is evident that after 2 years of treatment, patients in state 1 show a survival probability of approximately 95%, whereas those in state 2, state 3, and state 4 show survival probabilities of 65%, 30%, and 20%, respectively. Even after 4 years of treatment, patients in state 1 maintain a higher survival probability of about 65% with a gradual decline, in contrast to those in state 4, who show a much lower survival probability of about 5%, experiencing a rapid decline. These findings emphasize the efficacy of early treatment initiation, suggesting that initiating treatment at earlier stages of the disease leads to significantly better long-term outcomes in terms of survival.

From Table 9, it can be observed that 1 year from now, there is a high probability (67.08%) that patients in state 1 will remain in this state, indicating the effectiveness of ART in maintaining high CD4 levels for a majority of patients. However, there are probabilities of transitioning to lower CD4 states: 23.94% to state 2, 5.44% to state 3, and 1.59% to state 4. The probability of dying (state 5) from this initial state is relatively low at 1.96%, reflecting that patients with strong immune systems under ART are at a low risk of immediate mortality. For patients in state 2, there is a significant probability (53.37%) of remaining in this state, suggesting that ART continues to be relatively effective. However, the transition to lower states indicates a considerable risk: 24.79% to state 3 and 7.16% to state 4, with a notable probability of transitioning to death (14.56%). The very low probability of improving to state 1 (0.12%) suggests that once patients' CD4 counts fall below 500 cells/mm^3^, it is challenging to regain the higher count despite continued ART. Also, patients in state 3 have a nearly equal chance of remaining in the same state (48.83%) or progressing to death (49.33%). This near-equal probability emphasizes the critical nature of this stage, where immediate interventions might be necessary to prevent mortality. The chances of improving to higher CD4 states are minimal, with 0.52% for state 1 and 1.24% for state 2, indicating that as the disease progresses, recovery becomes increasingly difficult. In state 4, the probability of death is alarmingly high at 55.06%, and 44.94% of patients remain in this state. Once patients transition to death, they remain in this state with a probability of 1.0000, indicating no possibility of recovery.

From Table 10, it can be observed that 5 years from now, there will be a higher probability of death for patients across all states, particularly those in states 2 to 4. This suggests that despite being on treatment, patients in these CD4 ranges may still face significant health risks, particularly for those with lower CD4 cell counts. Hence, emphasizing the critical nature of monitoring and managing HIV patients even on treatment cannot be overlooked.

In Table 11, the hazard ratios for state transitions show wide confidence intervals, indicative of substantial uncertainty in these estimates. This suggests that TB coinfection does not impart a statistically significant effect on the hazard ratios for transitioning between states of HIV progression among patients undergoing treatment in this study.

As shown in Table 12, the *p* value of 0.195 indicates no significant difference between the two models, suggesting that the inclusion of TB as a covariate does not substantially improve the model fit.

## 4. Discussion

This study confirms earlier observations that ART significantly prolongs survival and improves health outcomes for HIV patients, as evidenced by the prolonged sojourn times in higher CD4 states (state 1 and state 2). This consistency emphasizes the robustness of ART in managing HIV/AIDS, aligning with studies by Shoko and Chikobvu [16], Agada et al. [11], and Pandey and Galvani [22], which also highlighted the effectiveness of ART in maintaining patients in favorable health states. However, contrasting findings were observed regarding transitions between states. While our study observed significant transitions from higher to lower CD4 states, the transition probabilities varied from those reported in similar studies [18]. For instance, our data showed a higher probability of transitioning from state 2 to state 3 compared to state 4, indicating a critical phase where patients are more likely to experience immune decline rather than severe immunosuppression.

In contrast to the literature suggesting a significant impact of TB coinfection on HIV progression [14], our study found no statistically significant association between TB coinfection and hazard ratios for state transitions. This finding contrasts with the broader epidemiological evidence but emphasizes the specific context of our study population, where ART management may have mitigated the expected impact of TB on disease progression. This discrepancy highlights the variability in outcomes across different populations and the need for context-specific interventions.

The implications of our findings emphasize the importance of continuous monitoring and tailored interventions for HIV patients on ART, particularly in regions like the Ashanti Region of Ghana. The longer sojourn times observed in higher CD4 states suggest that early initiation and consistent adherence to ART can significantly enhance patient outcomes, aligning with global health strategies aimed at achieving viral suppression and reducing transmission.

## 5. Conclusion

This study aimed to assess the progression of HIV patients on ART in sub-Saharan Africa, with a specific focus on TB coinfection. Using a multistate Markov model, we found that patients with higher CD4 counts (state 1) experienced better outcomes, spending more time in stable health states. In contrast, patients with lower CD4 counts (states 3 and 4) had significantly higher risks of mortality.

Interestingly, TB coinfection did not significantly alter these transition probabilities. This may be attributed to the effective management of both HIV and TB in the cohort, which could have mitigated the potential adverse impact of TB on disease progression. These findings highlight the importance of early ART initiation to maintain CD4 counts above 350 cells/mm^3^, improving patient survival and quality of life. Clinically, regular CD4 monitoring and timely interventions are essential to prevent progression to severe stages. Additionally, integrated care for HIV and TB coinfection remains crucial in optimizing patient outcomes, especially in high-risk regions.

## Figures and Tables

**Table 1:** Count of transitions observed between states.

From	To				
	1	2	3	4	5
1	284	121	0	0	21
2	0	232	148	43	56
3	1	1	0	0	40
4	0	0	0	0	22

**Table 2:** Sociodemographic characteristics of patients on ART treatment.

Variable	Frequency (%)
*Gender*	
Male	267 (64.18%)
Female	149 (35.82%)

*Age group (years)*	
≤ 45	93 (22.36%)
> 45	323 (83.17%)

*Religion*	
Christian	110 (26.44%)
Muslim	35 (8.41%)
Traditionalist	121 (29.09%)
Others	150 (36.06%)

*Marital status*	
Married	30 (7.21%)
Single	310 (74.52%)
Widowed	32 (7.69%)
Divorced	44 (10.58%)

*Occupation*	
Formally employed	42 (10.10%)
Self-employed	138 (33.17%)
Unemployed	127 (30.53%)
Others	109 (26.20%)

*Ethnicity*	
Akan	198 (47.60%)
Ewe	118 (28.37%)
Fafra	69 (16.59%)
Others	31 (7.45%)

*Tuberculosis status*	
Positive	15 (3.61%)
Negative	401 (96.39%)

*Hepatitis status*	
Positive	70 (16.83%)
Negative	346 (83.17%)

**Table 3:** Number of patients remaining alive and dead after treatment period.

Sex	Alive	Deceased	*χ* ^2^	*p* value
Female	79 (53.70%)	70 (47.60%)	18.266	0.001
Male	198 (74.40%)	69 (25.90%)		

**Table 4:** Number of patients who developed TB disaggregated by sex and when.

Sex	State	Frequency
Female	4	38 (61.30%)
Male	4	24 (38.70%)

**Table 5:** Transition intensities and their corresponding confidence intervals for the model.

(*ij*)	Intensities (*ω*)	95% CI	
*S*1-*S*1	−0.3996	−0.4716	−0.3386
*S*1-*S*2	0.3996	0.3386	0.4716
*S*1-*S*3	0.0000	0.0000	0.0000
*S*1-*S*4	0.0000	0.0000	0.0000
*S*1-*S*5	0.0000	0.0000	0.0000
*S*2-*S*1	0.0000	0.0000	0.0000
*S*2-*S*2	−0.6335	−0.7174	−0.5594
*S*2-*S*3	0.4873	0.4190	0.5666
*S*2-*S*4	0.1462	0.1077	0.1986
*S*2-*S*5	0.0000	0.0000	0.0000
*S*3-*S*1	0.0090	0.0013	0.0643
*S*3-*S*2	0.0235	0.0026	0.1922
*S*3-*S*3	−0.7228	−0.9047	−0.5775
*S*3-*S*4	0.0000	0.0000	0.0000
*S*3-*S*5	0.6914	0.5524	0.8655
*S*4-*S*1	0.0000	0.0000	0.0000
*S*4-*S*2	0.0000	0.0000	0.0000
*S*4-*S*3	0.0000	0.0000	0.0000
*S*4-*S*4	−0.7998	−1.1560	−0.5532
*S*4-*S*5	0.7998	0.5532	1.1560
*S*5-*S*1	0.0000	0.0000	0.0000
*S*5-*S*2	0.0000	0.0000	0.0000
*S*5-*S*3	0.0000	0.0000	0.0000
*S*5-*S*4	0.0000	0.0000	0.0000
*S*5-*S*5	0.0000	0.0000	0.0000

**Table 6:** Mean sojourn time in each state.

State	Estimates	SE	L	U
*S*1	2.5024	0.2114	2.1205	2.9531
*S*2	1.5785	0.1002	1.3938	1.7877
*S*3	1.3835	0.1584	1.1054	1.7315
*S*4	1.2503	0.2352	0.8647	1.8077

**Table 7:** Total length of stay.

State 1	State 2	State 3	State 4	State 5
2.5273	1.6331	1.1009	0.2986	Inf

**Table 8:** Probability of each state being next.

From	To				
	*S*1	*S*2	*S*3	*S*4	*S*5
*S*1	0.0000	1.0000	0.0000	0.0000	0.0000
*S*2	0.0000	0.0000	0.7692	0.2308	0.0000
*S*3	0.0125	0.0309	0.0000	0.0000	0.9566
*S*4	0.0000	0.0000	0.0000	0.0000	1.0000
*S*5	0.0000	0.0000	0.0000	0.0000	0.0000

**Table 9:** Probabilities of state transitions at time *t* = 1.

	*S*1	*S*2	*S*3	*S*4	*S*5
State 1	0.6708	0.2394	0.0544	0.0159	0.0196
*S*2	0.0012	0.5337	0.2479	0.0716	0.1456
*S*3	0.0052	0.0124	0.4883	0.0008	0.4933
*S*4	0.0000	0.0000	0.0000	0.4494	0.5506
*S*5	0.0000	0.0000	0.0000	0.0000	1.0000

**Table 10:** Probabilities of state transitions at time *t* = 5.

	*S*1	*S*2	*S*3	*S*4	*S*5
*S*1	0.1382	0.1657	0.1416	0.0383	0.5161
*S*2	0.0032	0.0492	0.0876	0.0222	0.8378
*S*3	0.0032	0.0066	0.0331	0.0017	0.9554
*S*4	0.0000	0.0000	0.0000	0.0183	0.9817
*S*5	0.0000	0.0000	0.0000	0.0000	1.0000

**Table 11:** Transition intensities with hazard ratios for tuberculosis coinfection.

	Baseline	Confidence interval		TB coinfection	Confidence interval	
		Lower	Upper		Lower	Upper
*S*1-*S*1	−0.4041	−0.4078	−0.3419			
*S*1-*S*2	0.4020	0.339	0.4767	0.8082	4.48E − 01	1.46E + 00
*S*1-*S*5	0.0021	0.0000	0.3863	0.5368	1.09E − 05	2.65E + 04
*S*2-*S*2	−0.6106	−0.7074	−0.5271			
*S*2-*S*3	0.4711	0.3897	0.5695	0.3971	3.15E − 02	5.01E + 00
*S*2-*S*4	0.1387	0.0717	0.2683	0.2289	1.13E − 06	4.65E + 04
*S*2-*S*5	0.0009	0.0000	0.1034	1.1225	9.36E − 16	1.35E + 15
*S*3-*S*1	0.0091	0.0000	0.0999	0.9886	3.07E − 14	3.18E + 13
*S*3-*S*2	0.0225	0.0019	0.259	0.9881	1.54E − 12	6.35E + 11
*S*3-*S*3	−0.7308	−0.9768	−0.5468			
*S*3-*S*5	0.6992	0.5208	0.9387	1.4239	2.27E − 02	8.95E + 01
*S*4-*S*4	−0.8166	−2.611	−0.2554			
*S*4-*S*5	0.8166	0.2554	2.6111	1.7998	2.16E − 10	1.50E + 10

**Table 12:** Likelihood ratio test comparing models with and without covariates.

Model comparison	Likelihood ratio statistic	Degrees of freedom	*p* value
Model with covariates (TB) versus model without covariates	3.69	2	0.195

## Data Availability

Data are available on request from the authors.
